# Analysis of Clinical Criteria for Discharge Among Patients Hospitalized for COVID-19: Development and Validation of a Risk Prediction Model

**DOI:** 10.1007/s11606-024-08856-x

**Published:** 2024-06-27

**Authors:** Jeffrey L. Schnipper, Sandra Oreper, Colin C. Hubbard, Dax Kurbegov, Shanna A. Arnold Egloff, Nader Najafi, Gilmer Valdes, Zishan Siddiqui, Kevin J. O.’Leary, Leora I. Horwitz, Tiffany Lee, Andrew D. Auerbach

**Affiliations:** 1 Hospital Medicine Unit, Division of General Internal Medicine and Primary Care, Brigham and Women’s Hospital, Boston, MA USA; 2 Harvard Medical School, Boston, MA USA; 3 Division of Hospital Medicine, University of California, San Francisco, San Francisco, CA USA; 4 COVID-19 Consortium of HCA Healthcare and Academia for Research Generation (CHARGE), Nashville, TN USA; 5 HCA Healthcare, Sarah Cannon Research Institute (SCRI), Nashville, TN USA; 6 HCA Healthcare, HCA Healthcare Research Institute (HRI), Kansas City, MO USA; 7 Division of Hospital Medicine, John Hopkins University School of Medicine, Baltimore, MD USA; 8 Division of Hospital Medicine, Northwestern University, Feinberg School of Medicine, Chicago, IL USA; 9 Department of Population Health, Department of Medicine, NYU Grossman School of Medicine; Center for Healthcare Innovation and Delivery Science, NYU Langone Health, New York City, NY USA

**Keywords:** COVID-19, hospital readmission, risk assessment

## Abstract

**Background:**

Patients hospitalized with COVID-19 can clinically deteriorate after a period of initial stability, making optimal timing of discharge a clinical and operational challenge.

**Objective:**

To determine risks for post-discharge readmission and death among patients hospitalized with COVID-19.

**Design:**

Multicenter retrospective observational cohort study, 2020–2021, with 30-day follow-up.

**Participants:**

Adults admitted for care of COVID-19 respiratory disease between March 2, 2020, and February 11, 2021, to one of 180 US hospitals affiliated with the HCA Healthcare system.

**Main Measures:**

Readmission to or death at an HCA hospital within 30 days of discharge was assessed. The area under the receiver operating characteristic curve (AUC) was calculated using an internal validation set (33% of the HCA cohort), and external validation was performed using similar data from six academic centers associated with a hospital medicine research network (HOMERuN).

**Key Results:**

The final HCA cohort included 62,195 patients (mean age 61.9 years, 51.9% male), of whom 4704 (7.6%) were readmitted or died within 30 days of discharge. Independent risk factors for death or readmission included fever within 72 h of discharge; tachypnea, tachycardia, or lack of improvement in oxygen requirement in the last 24 h; lymphopenia or thrombocytopenia at the time of discharge; being ≤ 7 days since first positive test for SARS-CoV-2; HOSPITAL readmission risk score ≥ 5; and several comorbidities. Inpatient treatment with remdesivir or anticoagulation were associated with lower odds. The model’s AUC for the internal validation set was 0.73 (95% CI 0.71–0.74) and 0.66 (95% CI 0.64 to 0.67) for the external validation set.

**Conclusions:**

This large retrospective study identified several factors associated with post-discharge readmission or death in models which performed with good discrimination. Patients 7 or fewer days since test positivity and who demonstrate potentially reversible risk factors may benefit from delaying discharge until those risk factors resolve.

**Supplementary Information:**

The online version contains supplementary material available at 10.1007/s11606-024-08856-x.

## BACKGROUND

Four years into the COVID-19 pandemic, there are still many unanswered questions about how best to care for patients affected by this disease. One important question is determining when it is safe to discharge patients from the hospital. Unlike other infectious diseases, early in the pandemic, it became clear that COVID-19 often got worse in the second week of illness after a period of initial stability.^1^ Discharging patients too soon can increase the risk that patients will get sicker after leaving the hospital, potentially leading to readmission, delays in care, and worse outcomes. But keeping patients in the hospital for an unnecessarily long time can mean overwhelming health care systems already exceeding full capacity, increasing the risk of iatrogenic complications, and creating financial and resource allocation strain on healthcare systems. It is thus critical to understand who can be discharged safely, minimizing length of stay and bed occupancy, while also minimizing post-discharge adverse outcomes such as readmission and death. Yet, we have demonstrated tremendous variability among health care systems in terms of clinical criteria for discharge.^2^

Observational studies on this subject have found associations between shorter hospital length of stay and readmission risk,^3–7^ suggesting that some patients were indeed discharged too soon. Other commonly found risk factors for readmission after COVID-19 have included comorbidities, older age, male sex, history of smoking, obesity, being febrile at discharge, and discharge to skilled nursing facilities or home health care services.^3–14^ Remdesivir treatment has been shown to be protective.^15^ However, most studies using detailed EHR-level data were too small to evaluate other risk factors such as vital signs other than temperature or inflammatory markers, and, to our knowledge, none compared adverse post-discharge outcomes in patients who met particular combinations of discharge criteria.

The goals of this study were to determine the risks for post-discharge adverse outcomes among patients hospitalized with COVID-19 respiratory disease, using a large, multi-center database; to develop a model to distinguish patients at high vs. low risk based on criteria present at the time of discharge; to internally and externally validate the model; and to compare its performance to other sets of published or commonly used criteria.

## METHODS

### Overview

We conducted a retrospective, multi-center, observational cohort study using the COVID-19 Consortium of HCA Healthcare and Academia for Research Generation (CHARGE) dataset.^16^ HCA Healthcare (Nashville, TN) is comprised of 185 hospitals mostly located in the Southern and Western United States. The CHARGE dataset was curated by personnel at HCA, Sarah Cannon (the cancer institute of HCA), and Genospace (Boston, MA) using clinical, billing, and administrative data from the electronic health record used at its sites (Meditech, Westwood, MA). Governance was provided by a steering committee with representation from HCA leadership, AHRQ, and ten partner academic institutions, including the Hospital Medicine Re-engineering Network (HOMERuN), a consortium of hospitalist leaders and researchers at academic medical centers throughout the USA. The study was approved by the WCG Institutional Review Board.

### Setting and Participants

Inclusion criteria consisted of the following: Hospitalized at an HCA Healthcare-affiliated hospital from March 2, 2020, through February 11, 2021; positive PCR test for SARS-CoV-2 no earlier than 14 days prior to hospital admission; age 18 years or older; and primary admitting or final diagnosis of COVID-19 respiratory disease using a validated set of diagnostic ICD-10 codes.^16^

Exclusion criteria were nosocomial COVID-19 (first positive test > 7 days after admission); discharged against medical advice, with hospice care, or to another acute care hospital; expired during the hospitalization; admission to a non-Meditech hospital; elective or otherwise ineligible index admission (e.g., cancelled); encounters with missing diagnosis codes or discharge disposition; and discharged less than 30 days before February 11, 2021 (i.e., incomplete follow-up data).

### Outcomes

The primary outcome was readmission to or hospital death at any HCA hospital within 30 days of discharge. We did not count readmissions to rehabilitation or psychiatric facilities or elective readmissions (but they would count as a death if a patient died during or subsequent to that event).

### Predictors and Potential Confounders

Candidate predictors (see Appendix Table 4) included covariates generally known to be associated with readmission and ones unique to COVID-19, including patient demographics; specific comorbidities and van Walraven-Elixhauser comorbidity score;^17^ fever status in the 72 h prior to discharge; respiratory rate, heart rate, and blood pressure in the 24 h prior to discharge; worst oxygenation requirement in the last 24 and 72 h prior to discharge; worst and last laboratory values during hospitalization; inpatient complications of COVID-19; COVID-19 treatments; other treatments (e.g., anticoagulation at prophylactic or treatment doses); admission source; discharge disposition; days since first positive SARS-CoV-2 test; ICU stay during hospitalization; and time period of the pandemic by quarter. Potential confounders included the HOSPITAL score, a predictive tool for 30-day, potentially avoidable readmissions established and validated in 4 countries by our team.^18,19^ See Appendix Table 4 for categorization and proportion of missing data for each variable.

### Development and Validation of the Model

Patients who suffered the primary outcome were compared to those who did not with respect to several demographic and clinical variables using descriptive statistics. The cohort was randomly divided into training (two-thirds of the eligible HCA cohort; *n* = 40,847) and internal validation (*n* = 21,347) sets. Missing categorical variables were imputed with the mode or “missing” was added as a category level, depending on the degree of missingness (see Appendix Table 4), and continuous variables were imputed with the median. To create a clinically meaningful and interpretable subset of candidate predictors, we used a two-step procedure. First, we fit a logistic model on the training data using least absolute shrinkage and selection operator (LASSO) regression.^20^ We used tenfold cross-validation with deviance as the loss to select the optimal regularization penalty tuning parameter (lambda). We chose the largest value of lambda with a deviance within one standard error of the minimum to prioritize parsimony.^20^ We then fit a logistic regression model using unpenalized maximum likelihood to the training data set using the variables selected in the LASSO regression to report adjusted odds ratios and confidence intervals for each variable.^21^ To obtain unbiased estimates of the confidence intervals in this final model, we employed multiple imputation (using the MICE package in R)^22^ for any remaining continuous variables by creating ten multiply-imputed data sets, using predictive mean matching and Rubin’s rules to combine parameter estimates and derive standard errors. In these models, we added a small set of interaction terms based on subgroup analyses to look for effect modification: by discharge destination (home, SNF/Rehab/LTAC, other or missing), respiratory rate at discharge, receipt of remdesivir, receipt of corticosteroids, and pandemic quarter.

To evaluate the predictive performance of our model, we calculated the area under the receiver operating characteristic (ROC) curve (AUC) for the training data set and the remaining one-third of the CHARGE cohort (internal validation). We also produced calibration diagrams for the internal validation cohort. We identified a high-risk and low-risk cohort for our outcome using the cutoff of risk that maximized the product of sensitivity and specificity using the cutpointr package in R.^23^ To externally validate our model, we collected comparable variables from March 2 to December 31, 2020, from seven hospitals in the HOMERuN consortium who agreed to provide their data, including Brigham and Women’s Hospital, BWH Faulkner Hospital (BWH’s community affiliate), Massachusetts General Hospital, University of California San Francisco Medical Center, Northwestern Memorial Hospital, Johns Hopkins Hospital, and NYU Langone Health. Johns Hopkins Hospital could not provide an EHR indicator for ICU admission (because of the conversion of general wards to temporary ICUs); therefore, the initiation and discontinuation of mechanical ventilation or ECMO was used as a proxy for ICU admission and discharge, respectively.

Lastly, we compared the model developed by our analyses with discharge criteria from the published literature or available online from hospitals in the HOMERuN network. Comparators included guidelines from CDC, University of Michigan, Johns Hopkins, Levine et al.,^24^ and a clinical gestalt. The guidelines for these criteria are included in Appendix Table 5. Using the CHARGE internal validation dataset, we compared the proportion of patients determined to be high risk, the risk of readmission or death in the high-risk vs. low-risk cohorts, the diagnostic odds ratio (and 95% confidence intervals), and the likelihood ratio positive and negative (and 95% confidence intervals) for being high risk and low risk, respectively.

Analyses were conducted using SAS v.9.4 (SAS Institute, Cary, NC) and R v.4.1.3 (R Foundation for Statistical Computing, Vienna, Austria). Unless otherwise stated, two-sided *p*-values < 0.05 were considered significant.

## RESULTS

**Figure 1:**
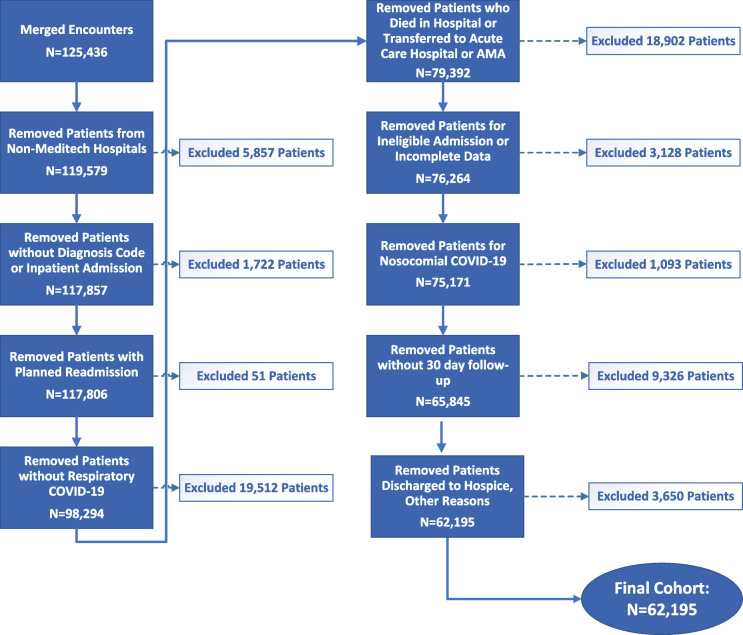
Study flow diagram.

Figure 1 illustrates the flow diagram. The original cohort consisted of 125,436 merged hospital encounters, while the final cohort, after data cleaning and exclusions, resulted in 62,195 patients. Most patients were excluded due to the absence of COVID-19 respiratory disease; died in the hospital, transferred to another acute hospital, or discharged against medical advice; or did not have 30 days of post-discharge follow-up by the dataset cutoff date. Mean age of the cohort was 61.9 years, and 51.9% were male. In this cohort, 4704 (7.6%) were readmitted or died within 30 days of discharge (7.2% were readmitted, 1.6% died). Characteristics of those patients who were readmitted or died within 30 days of discharge compared to those who did not are shown in Table 1. Those who suffered the primary outcome were generally older, more likely to be male, to be White, to be non-Hispanic, to be a former smoker, to have a shorter length of stay, to be discharged to a destination other than home, to be with a COVID-19 complication of CHF or AKI in the hospital, and less likely to receive remdesivir.

**Table 1:** Patient Characteristics

	Readmission or death within 30 days				
	No		Yes		
Total cohort = 62,195	*N* = 57,491 (92.4%)		*N* = 4,704 (7.6%)		*P* value
Age, years					< 0.001
Mean (SD)	61.3	(16.8)	68.8	(15.5)	
Sex					< 0.001
Male	29,637	(51.6%)	2642	(56.2%)	
Race					< 0.001
White	32,256	(56.1%)	2902	(61.7%)	
Black	10,817	(18.8%)	863	(18.3%)	
Asian	1790	(3.1%)	133	(2.8%)	
Other or NA	12,628	(22.0%)	806	(17.1%)	
Ethnicity					< 0.001
Hispanic	17,323	(30.1%)	1164	(24.7%)	
Smoking status					
Current	210	(0.4%)	23	(0.5%)	0.18
Former	10,025	(17.4%)	1109	(23.6%)	< 0.001
Admission source					< 0.001
Facility	1419	(2.5%)	156	(3.3%)	
Referral	48,922	(85.1%)	3889	(82.7%)	
Other or NA	7150	(12.4%)	659	(14.0%)	
Length of stay					< 0.001
Median (IQR)	5.3	(3.0, 9.3)	4.2	(2.0, 8.1)	
Discharge destination					< 0.001
Home	45,577	(79.3%)	3156	(67.1%)	
SNF/Rehab/LTAC	8539	(14.9%)	1120	(23.8%)	
Other or NA	3375	(5.9%)	428	(9.1%)	
COVID complications					
VTE	1924	(3.3%)	128	(2.7%)	0.021
CHF	7428	(12.9%)	1098	(23.3%)	< 0.001
DIC	88	(0.2%)	4	(0.1%)	0.24
AKI	14,205	(24.7%)	1551	(33.0%)	< 0.001
COVID treatments					
Convalescent plasma	17,266	(30.0%)	1116	(23.7%)	< 0.001
IL-6 inhibitors	988	(1.7%)	65	(1.4%)	0.085
JAK inhibitors	12	(0.0%)	0	(0.0%)	> 0.999
Corticosteroids	45,278	(78.8%)	3777	(80.3%)	0.013
Remdesivir	23,755	(41.3%)	1370	(29.1%)	< 0.001

*SNF* skilled nursing facility, *LTAC* long-term acute care, *VTE* venous thromboembolism, *CHF* congestive heart failure, *DIC* dessiminated intravascular coagulation, *AKI* acute kidney injury, *IL-6* interleukin 6, *JAK* janus kinase

In the final multivariable logistic regression model (Table 2), several factors were independently associated with 30-day readmission or death. These included fever within 72 h of discharge; tachypnea, tachycardia, or use of supplementary oxygen in the 24 h prior to discharge; lack of improvement in oxygen requirement (i.e., worst requirement in the last 24 h no better than the worst requirement in the 72 h prior to discharge); lymphopenia or thrombocytopenia at the time of discharge; Elixhauser comorbidity score of 6 or greater; current or former smoker; history of hypertension, chronic obstructive pulmonary disease (COPD), diabetes mellitus, chronic kidney disease, cardiovascular disease, or cancer; hospitalization complicated by heart failure; discharge ≤ 7 days since first positive test for SARS-CoV-2; age 50 or older (compared with age 18–39); male sex; treatment with corticosteroids any time during the hospitalization; discharge destination other than home; and HOSPITAL readmission risk score 5 or greater. Inpatient treatment with remdesivir and prophylactic or treatment-dose anticoagulation were associated with lower odds, as were number of days in the intensive care unit (ICU) and discharge directly from ICU. Notably non-significant factors included BMI and improvement or normalization of inflammatory markers such as C-reactive protein, d-dimer, ferritin, lactate dehydrogenase, and troponin. Using the beta-coefficients from Table 2, the odds of death or readmission in a given patient can be calculated as e^[−4.53+ ß1(fever status) + ß2(respiratory rate) + ß3(Heart Rate) + …]^, and the probability of death or readmission is odds/(1 + odds) (see Supplementary Material for an Excel spreadsheet to calculate risk).

**Table 2:** Independent Risk Factors for Primary Outcome in Logistic Regression

Predictor	Beta coefficient*	Adjusted OR (95% CI)
Fever status (vs. afebrile and no antipyretics)		
Afebrile in last 72 h but on antipyretics	0.11015	1.12 (1.02–1.22)
Febrile in last 72 h	0.56408	1.76 (1.57–1.97)
RR > 24 in last 24 h	0.21388	1.24 (1.05–1.46)
Heart rate > 100 in last 24 h	0.31765	1.37 (1.24–1.52)
Worst O_2_ requirement last 24 h (vs. room air)		
Low flow suppl. O_2_	0.14343	1.15 (1.05–1.27)
High flow suppl. O_2_	0.25227	1.29 (1.01–1.64)
CPAP/BiPAP	0.77072	2.16 (1.62–2.89)
Vent or ECMO	1.48747	4.43 (2.82–6.95)
Missing	0.47786	1.61 (1.35–1.92)
O_2_ requirement last 24 h not better than last 72 h or missing	0.14135	1.15 (1.04–1.28)
Discharge absolute lymphocyte count (vs. > 800)		
≤ 800	0.36717	1.44 (1.29–1.61)
Missing	0.20476	1.23 (1.10–1.37)
Last platelet count (vs. normal)		
Low	0.46992	1.60 (1.42–1.81)
High	− 0.26073	0.77 (0.63–0.94)
Missing	0.52145	1.68 (1.49–1.90)
Elixhauser-Walraven Comorbidity Score (vs. < 0)		
0–5	0.00616	1.01 (0.89–1.14)
6–12	0.16771	1.18 (1.04–1.34)
13 +	0.22677	1.25 (1.08–1.46)
Current/former smoker	0.14101	1.15 (1.05–1.26)
History of hypertension	0.15066	1.16 (1.04–1.30)
History of chronic pulmonary disease	0.08824	1.09 (1.00–1.20)
History of diabetes mellitus	0.11991	1.13 (1.04–1.22)
History of renal failure	0.26468	1.30 (1.18–1.44)
History of CAD, PVD or MI	0.14956	1.16 (1.06–1.27)
History of cancer	0.22991	1.26 (1.04–1.53)
CHF (as complication of COVID)	0.10326	1.11 (0.99–1.24)
Days since 1st ( +) COVID test (vs. ≤ 7)		
8–10	− 0.20959	0.81 (0.71–0.92)
11–14	− 0.41971	0.66 (0.56–0.77)
> 14	− 0.37473	0.69 (0.59–0.80)
Days in ICU (per additional day)	− 0.05982	0.94 (0.93–0.96)
Direct ICU discharge	− 1.09720	0.33 (0.24–0.47)
Age, years (vs. 18–39)		
40–49	0.02783	1.03 (0.82–1.29)
50–64	0.45942	1.58 (1.31–1.92)
65–74	0.75787	2.13 (1.75–2.60)
75–84	0.94104	2.56 (2.09–3.14)
85 +	1.01371	2.76 (2.22–3.43)
Male sex	0.19577	1.22 (1.12–1.32)
Receipt of remdesivir	− 0.29566	0.74 (0.67–0.82)
Receipt of corticosteroids	0.47822	1.61 (1.42–1.83)
Anticoagulants (v. none received)		
Prophylaxis dose	− 0.18774	0.83 (0.71–0.97)
Treatment dose	− 0.21220	0.81 (0.74–0.89)
Discharge destination (vs. home)		
SNF/Rehab/LTAC	0.28975	1.34 (1.09–1.64)
Other** or missing	0.83037	2.29 (1.78–2.96)
HOSPITAL readmission risk score (vs. < 5)		
5–6	0.34803	1.42 (1.20–1.67)
7 +	0.70195	2.02 (1.47–2.76)
RR > 24 in last 24 h X receipt of remdesivir	0.31301	1.37 (1.04–1.79)
Receipt of corticosteroids X discharge to Other** or missing discharge destination	− 0.48756	0.61 (0.45–0.84)
Receipt of corticosteroids X discharge to SNF/Rehab/LTAC	0.08616	1.09 (0.87–1.36)

*RR* respiratory rate, *O*_*2*_ supplementary oxygen, *CPAP* continuous positive airway pressure, *BiPAP* Bi-level positive airway pressure, *ECMO* extra-corporeal membrane oxygenation, *CAD* coronary artery disease, *PVD* peripheral vascular disease, *MI* myocardial infarction, *CHF* congestive heart failure, *ICU* intensive care unit, *SNF* skilled nursing facility, *LTAC* long-term acute care

^*^Y-intercept =  − 4.53108

^**^Includes shelter, prison, or congregate living facility (e.g., that provides custodial or other supportive care)

Out of a select few chosen interaction terms identified by subgroup analyses, the only two that were significant were abnormal respiratory rate at discharge by receipt of remdesivir (higher odds of primary outcome than otherwise expected from the two terms individually) and receipt of corticosteroids by discharge to Other location (shelter, prison, or congregate living facility) (lower odds than expected).

**Figure 2:**
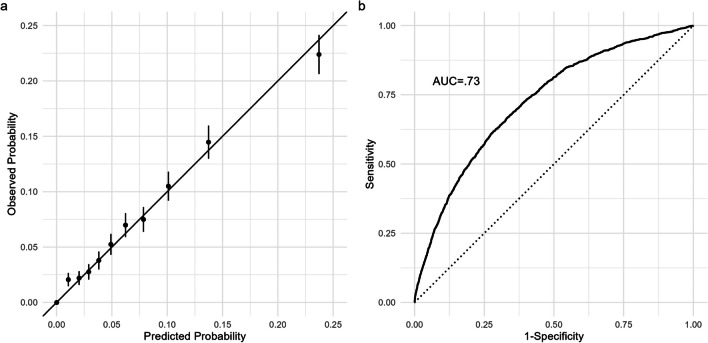
**a** Model calibration plot for internal validation cohort. **b** Receiver operating characteristic (ROC) curve for internal validation cohort.

The model was well calibrated, as the observed and expected 30-day mortality or re-admission rates in the validation data were highly correlated. Calibration was less precise at the lowest decile (2.1% observed vs. 1.1% predicted) and highest decile (22.4% observed vs. 23.7%) compared with the middle deciles of risk (Fig. 2a). The model discrimination as measured by AUC in the derivation set was 0.75 (95% confidence interval (CI) 0.74–0.76). In the internal validation set (*N* = 21,347), the AUC was 0.73 (95% CI 0.71–0.74) (Fig. 2b). Using the cutoff of risk (7.8%) that maximized the product of sensitivity and specificity, the model distinguished between 7464 high-risk patients (35% of the internal validation cohort) with a 14.6% probability of readmission or death (i.e., positive predictive value) from 13,883 low-risk patients (65% of the internal validation cohort) with a 4.1% probability of readmission or death (i.e., negative predictive value of 95.9%). The sensitivity of the model was 65%, specificity 68%, and accuracy 67%.

In the external validation cohort (*N* = 11,338), 8.7% were readmitted, 0.7% died, and 9.0% were readmitted or died. Compared to the CHARGE cohort, the external validation cohort was younger, with more current smokers, fewer complications of CHF and AKI, and less use of remdesivir and corticosteroids (Appendix Table 6). The AUC of our model in the external validation cohort was 0.66 (95% CI 0.64–0.67). In this cohort, the model could distinguish between 21% of the patients as high risk, with 16.8% probability of readmission or death, and the remaining 79% of patients with a 6.9% probability of readmission or death. As shown in Table 3, using the CHARGE cohort, our model (HCA) outperformed the other models it was tested against. Specifically, the likelihood ratio for the primary outcome of being high risk in our model was 2.02 (95% CI 1.94–2.10), while it varied from 1.02 to 1.16 for the other models.

**Table 3:** Comparison of Clinical Criteria for Discharge

Criteria	% of cohort defined as high risk	Readmission or death in high-risk patients (PPV)	Readmission or death in low-risk patients (1 – NPV)	Diagnostic OR (95% CI)	LR positive (95% CI)	LR negative (95% CI)
CDC	97.6%	7.9%	2.9%	2.83 (1.69–4.75)	1.02 (1.01–1.02)	0.36 (0.22–0.60)
Michigan	56.3%	8.3%	5.8%	1.46 (1.27–1.69)	1.16 (1.11–1.22)	0.80 (0.73–0.87)
Johns Hopkins	81.2%	7.7%	6.5%	1.19 (1.03–1.38)	1.03 (1.01–1.06)	0.86 (0.77–0.98)
Levine et al.^24^	95.7%	7.8%	5.3%	1.52 (1.13–2.04)	1.02 (1.01–1.02)	0.67 (0.50–0.89)
Clinical Gestalt	61.2%	8.4%	5.9%	1.45 (1.27–1.67)	1.14 (1.09–1.19)	0.78 (0.71–0.86)
HCA	35.0%	14.6%	4.1%	3.94 (3.55–4.38)	2.02 (1.94–2.10)	0.51 (0.48–0.55)

*CDC* Centers for Disease Control and Prevention 2020 criteria for removal of isolation precautions, *HCA* HCA Healthcare Model (i.e., model derived from this study), *PPV* positive predictive value, *NPV* negative predictive value, *LR* likelihood ratio

## DISCUSSION

In this large, multi-center retrospective cohort study of patients hospitalized with COVID-19 respiratory disease during the first year of the pandemic, 7.6% of patients were readmitted or died within 30 days of discharge. Independent predictors of the primary outcome included a variety of demographic and clinical factors present at the time of discharge. Our model had moderate discrimination when internally validated, fair discrimination when externally validated, and performed better than other criteria that have been recently published or were in common use in hospitals in our consortium.

The proportion of readmission or death within 30 days in our cohort is consistent with other COVID-19 studies,^7,10,25^ including a meta-analysis that found a 30-day readmission rate of 9.0% and mortality rate of 7.9%.^26^ These rates are lower than the readmission rates seen in typical medical patients.^27^ We hypothesize several reasons for this finding: COVID-19 patients tended to have less bio-psychosocial complexity than typical medical patients and longer lengths of stay, and once patients recovered from COVID-19 respiratory disease and/or were past their first week of illness, they tended not to have recrudescent disease. This hypothesis may also explain why days in the ICU and discharge directly from the ICU were associated with lower risk. It is also notable that shorter length of stay was associated with higher risk, which has been noted in other studies, and again lends strength to the hypothesis that some patients with COVID-19 were indeed discharged too soon. In almost every other observational study of medical patients, higher length of stay is associated with *increased* risk of readmission because both are markers of clinical severity.^19,28^ For this positive association to be reversed in the COVID-19 cohort, that is, to overcome confounding by severity of illness, suggests that the negative effect of premature discharge might be quite large. Our study also lends support to the observation that patients discharged in the first week of illness can deteriorate later in their course, at least during the first year of the pandemic in an immune-naïve population and prior to variant evolution. Patients discharged to facilities instead of home also had a higher risk of readmission or death; this association has been shown in other cohorts with^4,7^ and without COVID-19^29^ and is likely because discharge to facilities other than home is a surrogate marker of frailty.

It is notable that patients who received prophylactic or treatment dose anticoagulation were at lower risk for readmission or death. The effect of prophylactic vs. treatment-dose anticoagulation on non-critically ill hospitalized patients with COVID-19 has been controversial, with some studies (REMAP-CAP, ACTIV-4a, ATTACC, HEP-COVID)^30,31^ showing benefit of therapeutic dosing, and others (ACTION)^32^ not. These studies did not include readmission as an outcome. Our study lends support to the hypothesis that anticoagulation might be beneficial in preventing poor post-discharge outcomes in patients with COVID-19, but this would require further and longer-term investigation. It is also notable that receipt of remdesivir was associated with lower risk of poor outcomes (as has been noted in other studies)^15^ but that receipt of corticosteroids was observed to have the opposite effect (also shown in other studies),^11^ even after adjustment for several risk factors. It is biologically plausible that stopping anti-inflammatory medications such as dexamethasone at the time of discharge, as is commonly done, could lead to recrudescence of disease, but we cannot also rule out residual confounding by indication (i.e., patients perceived as sicker by their clinicians received steroids, based on unmeasured factors such as shortness of breath, abnormal findings on lung exam, and abnormal findings on chest imaging).

Most of the clinical factors associated with readmission or death were not surprising, but it was notable that none of the inflammatory markers we tested were significant in our models. We introduced these variables into the models in several ways, including absolute levels at discharge and relative improvement during the hospitalization, but none of these influenced the results. It may also be that the amount of missing data for these fields limited our ability to find significant effects. BMI also did not matter despite being a known risk factor for severe disease at the time of admission; BMI may be collinear with other comorbidities that have a greater association with risk. Race and ethnicity were associated with outcome in bivariable analyses, possibly reflecting differences in exposure to COVID-19 and access to care, but they were not significant after multivariable adjustment.

This model could be used to influence decision-making as a patient nears time for hospital discharge. For example, high-risk patients with modifiable risk factors (e.g., lack of improvement in oxygen requirement, fever, tachypnea, or tachycardia) are worthy of consideration for delaying discharge until these risk factors resolve, whereas high-risk patients with non-modifiable factors (e.g., age, comorbidities, nosocomial complications) may be considered for discharge to a more monitored post-acute setting or enrollment in a home monitoring program.^33–35^ Both of these strategies, and use of our model, are worthy of evaluation in prospective studies.

We used a cutoff for high risk that maximized the product of sensitivity and specificity in our model. One might argue that to prevent an outcome such as readmission or death, a cutoff that favors sensitivity might be preferable. However, a 4.1% probability in the low-risk cohort is already quite low compared with typical medical inpatients. Using a lower cutoff would reduce the risk in the low-risk cohort but at the cost of identifying more patients at high risk, potentially prolonging length of stay in these patients.

An important question is the relevance of this model, based on data from the first year of the pandemic, in the context of the evolving epidemiology of COVID-19. Many of the predictive factors are simply markers of clinical instability or comorbidity and are unlikely to have changed substantially. At least one study, from the UK, showed the continued relevance of risk scores generated during the first wave of the pandemic to predict outcomes during the second wave.^12^ The main unanswered question is the extent to which patients can still deteriorate after a period of initial improvement. There are emerging (unpublished) data that recrudescence after the first week of illness is currently rare but still present in patients who are immunocompromised, especially on rituximab and other B cell–depleting treatments, where there is evidence that the period of viremia is prolonged.^36,37^ There is also the phenomenon of rebound after treatment with oral antiviral treatment such as nirmatrelvir/ritonavir, but these events are relatively transient and mild.^38–40^

The results of our study are consistent with prior studies of readmission after COVID-19 hospitalization^41,42^ but also adds to what is currently known. Several studies have suggested that being febrile at discharge might be a risk factor; our study strongly supports that finding. Somani et al. found a trend towards less anticoagulation as a risk factor,^6^ again something our study more strongly supports. Other studies have also found the association with discharge to destinations other than home as a risk factor.^4,7,14^ Our study, due to its size, identifies several additional risk factors not previously established, including tachycardia and lack of improvement in oxygen requirement in the 72 h prior to discharge, and the predictive value of the HOSPITAL score, which has been shown to be useful in general medical patients but has not previously been shown to be useful in patients with COVID-19.

The results of this study should be viewed in light of its limitations. While large and diverse, this cohort is from one health care system (mostly community hospitals in the Southern US), which could limit its generalizability (and the model performed less well when externally validated in other health care systems). The analysis suffers from missing data, especially for some laboratory values, that might have limited our ability to find significant effects, as noted above. Missing data was particularly an issue with the external validation cohort, which may partly explain the poorer performance of the model in that cohort; another possible explanation are differences in demographics and in the use of treatments for COVID-19 in the two cohorts. Specifically, the external validation cohort consisted of academic medical centers, which likely cared for patients with higher comorbidity and where different factors may play a role in readmission risk. The study is also limited by the types of variables that could be collected; e.g., it did not include the presence of social support systems that can impact readmission risk.^14^ Outcome assessment may also have been incomplete, e.g., deaths not known to the health systems in the cohort, which could have limited the discrimination of our model. This current analysis only covers the first year of the pandemic; changes in the standard of care (e.g., in the use of dexamethasone), vaccination, and in the nature of the newest strains of SARS-CoV-2 could change these results, as noted above.

We also did not measure contextual factors, like the effects of periodic surges of patients with COVID-19 on bed capacity, that could have influenced outcomes.^43^ On the other hand, to our knowledge, this is one of the largest studies to evaluate post-discharge outcomes in the USA using detailed, EHR-level data.

In conclusion, in one of the largest retrospective studies to be conducted on this subject, we identified several factors that were associated with post-discharge readmission or death with moderate discrimination and good calibration, many of which could influence clinical decision-making at the time of discharge in patients hospitalized for COVID-19.

## Electronic supplementary material

Below is the link to the electronic supplementary material.

Supplementary file1 (XLSX 126 KB)

## Data Availability

Due to waiver of patient consent and restrictions of the CHARGE dataset, individual patient data cannot be shared. The data dictionary and statistical/analytic code can be made available upon reasonable request.

## Appendix

Tables 4, 5, 6

**Table 4:** Candidate Predictors in Penalized Logistic Regression Model (LASSO) and Predictors in Unpenalized Logistic Regression Prediction Model

				HCA training cohort *N* = 40,847	HCA validation cohort *N* = 21,347	HOMERuN validation cohort *N* = 11,338
Category	Predictor	Levels (if factor)	Imputed value^a^	Missing (%)	Missing (%)	Missing (%)
Demographic						
	Age, years	18–39, 40–49, 50–64, 65–74, 75–84, 85 +		0.0%	0.0%	0.0%
	Sex	Female, male		0.0%	0.0%	0.0%
	Race	White, Black, Asian, Hispanic, Other or Missing	Other or missing	3.5%		
	Hispanic ethnicity	Yes, No		0.0%		
	BMI	< 20, 20–25, 25–30, 30 + , Missing	Missing	23.1%		
	State-level Area Deprivation Index ranking	1–3, 4–5, 6–7, 8–9, 10, Missing	Missing	20.7%		
Medical/social history						
	Current/former smoker	Yes, no	No	12.8%	14.7%	0.0%
	History of hypertension	Yes, no	No	0.0%	0.0%	8.6%
	History of chronic pulmonary disease	Yes, no	No	0.0%	0.0%	8.6%
	History of diabetes mellitus	Yes, no	No	0.0%	0.0%	8.6%
	History of renal failure	Yes, no	No	0.0%	0.0%	8.6%
	History of coronary artery disease, peripheral vascular disease or myocardial infarction	Yes, no	No	0.0%	0.0%	7.5%
	History of congestive heart failure	Yes, no		0.0%		
	History of cancer	Yes, no	No	0.0%	0.0%	8.6%
	History of autoimmune disease	Yes, no		0.0%		
	Pre-admission immunosuppresion	Yes, no		0.0%		
Disease severity/readmission predictive scores						
	Elixhauser-Walraven score	≤ 0, 1–5, 6–11, 12 +	1–5	0.0%	0.0%	8.6%
	HOSPITAL score	0–4, 5–6, 7 +		0.0%	0.0%	0.0%
	WHO score on admission	0–6	0	27.5%		
	Maximum WHO score during hospitalization	0–6	0	2.1%		
Vital signs/O_2_ requirements						
	Febrile in last 72 h	Afebrile no antipyretics, afebrile and antipyretics, febrile		0.0%	0.0%	0.0%
	Hemodynamic stability last 24 h (SBP ≥ 90)	Yes, no	Yes	0.5%		
	Respiratory rate normal in last 24 h (RR ≤ 24)	Yes, no	Yes	0.7%	0.7%	0.2%
	Normal heart rate last 24 h (HR ≤ 100)	Yes, no	Yes	0.5%	0.5%	0.1%
	Worst O_2_ requirement last 24 h	Room air, Low flow suppl O_2_, High flow suppl O_2_, CPAP/BiPAP, ventilator or ECMO, missing	Missing	3.7%	4.3%	17.8%
	O_2_ requirement last 24 h lower than in last 72 h	O_2_ req. worse, O_2_ req. not better or NA	O_2_ req. not better or NA	3.7%	4.3%	17.8%
Laboratory						
	Discharge absolute lymphocyte count > 800	Yes, no, missing	Missing	42.2%	43.2%	29.9%
	Minimum absolute lymphocyte count during admission		Median	26.6%		
	Last absolute lymphocyte count prior to discharge		Median	42.2%	43.2%	29.9%
	Last C-reactive protein prior to discharge		Median	61.1%		
	Maximum C-reactive protein during admission		Median	39.5%		
	Last d-dimer prior to discharge		Median	70.4%		
	Maximum d-dimer during admission		Median	52.5%		
	Last ferritin prior to discharge		Median	70.1%		
	Maximum ferritin during admission		Median	44.4%		
	Last lactate dehydrogenase prior to discharge		Median	77.2%		
	Maximum lactate dehydrogenase during admission		Median	56.8%		
	Last troponin prior to discharge (as percentage of ULN)	Low, normal, high, none recorded	Median	90.8%		
	Last procalcitonin prior to discharge		Median	96.7%		
	Maximum procalcitonin during admission		Median	90.7%		
	Last albumin prior to discharge		Median	34.2%		
	Minimum albumin during admission		Median	18.7%		
	Last platelet count prior to discharge	Low, normal, high, none recorded	Median	21.3%	20.9%	0.3%
COVID-19 complications						
	Acute kidney injury	Yes, no		0.0%		
	Venous thromboembolism	Yes, no		0.0%		
	Disseminated intravascular coagulation (as complication of COVID-19)	Yes, no		0.0%		
	Congestive heart failure	Yes, no		0.0%	0.0%	0.0%
Treatment						
	Administration of remdesivir	Yes, no	No	0.0%	0.0%	0.1%
	Administration of corticosteroids	Yes, no		0.0%	0.0%	0.0%
	Administration of janus kinase (JAK) or interleukin (IL)-6 inhibitors	Yes, no		0.0%		
	Convalescent plasma	Yes, no		0.0%		
	Dose of anticoagulants received	None, prophylaxis, treatment		0.0%	0.0%	0.0%
	Administration of vasopressors	Yes, no		0.0%		
	Administration of opioids	Yes, no		0.0%		
	Administration of azithromycin	Yes, no		0.0%		
Other patient-level variables						
	Admission source	Home, facility, referral, other or missing	Other or missing	12.5%	0.0%	
	Discharge disposition	Home, SNF/rehab/LTAC, other or missing		0.0%	0.0%	0.0%
	Days since 1st ( +) SARS-CoV-2 test	0–7, 8–10, 11–14, > 14	0–7	0.1%	0.1%	6.1%
	Days in ICU			0.0%	0.0%	0.0%
	Direct ICU discharge	Yes, no		0.0%	0.0%	0.0%
	Pandemic quarter of admission date	Mar.–May 2020, Jun.–Aug. 2020, Sep.–Nov. 2020, Dec. 2020–Feb. 2021		0.0%		

Percentage missing for variables that were not selected in the final prediction model are not shown for the validation cohorts

^a^For categorical variables, the mode was imputed if missingness was rare (< 1%); otherwise, a separate category of “missing” was created

**Table 5:** Comparison Discharge Criteria

Rule	Criteria	
CDC guidelines for removal of isolation precautions	Afebrile and not on antipyretics for 72 h prior to discharge; and no supplemental oxygen, respiratory rate ≤ 20, and oxygenation by pulse oximetry ≥ 94% in the 24 h prior to discharge; and 7 or more days since first positive SARS-CoV-2 test	
University of Michigan criteria for discharge	Age < 70, normal c-reactive protein (CRP), no Elixhauser comorbidities, and no supplemental O_2_ in the last 24 h prior to discharge **or**	
	Oxygen requirement in the last 24 h less than on admission and < 2L by nasal cannula, and > 12 days from first positive COVID test **or**	
	LOS ≥ 48 h; vital signs normal (systolic blood pressure ≥ 90, heart rate ≤ 100, respiratory rate ≤ 24), and no supplemental oxygen in the last 24 h prior to discharge	
Johns Hopkins criteria for discharge (simplified)	No comorbidities (or 1–2 comorbidities and ≥ 10 days since first positive COVID test and never hypoxic), vital signs normal (as above), and no supplemental oxygen last 24 h prior to discharge, laboratory values (CRP, LDH, d-dimer, troponin, ferritin, lymphocyte count, procalcitonin, albumin), normal or improved (≥ 20% better if present) at discharge compared with admission **or**	
	1–2 comorbidities (or 3 or more comorbidities and ≥ 10 days since first positive COVID test and never hypoxic), vital signs normal last 24 h prior to discharge, no supplemental oxygen last 48 h prior to discharge, labs normal or improved at discharge compared with admission **or**	
	≥ 14 days after first positive COVID test	
Scoring system created by Levine and colleagues to predict poor outcomes after Emergency Department or Hospital discharge (score > 30: low risk)^24^	Characteristic	Score
	Age, years	
	18–45	5
	46–59	2
	60–73	1
	> 73	0
	Oxygen saturation, %, last value	
	< 94	0
	94–96	9
	97–98	14
	> 98	21
	Albumin, g/dL, last value	
	< 2.8	0
	2.8–3.3	5
	3.4–3.7	15
	> 3.7	29
Clinical gestalt	Worst oxygen requirement in the last 24 h prior to discharge better than the worst requirement during the last 72 h and 2L nasal cannula or less; **or**	
	No oxygen requirement in the last 24 h prior to discharge

**Table 6:** Comparison of Internal (CHARGE) and External (HOMERuN) Cohorts

	CHARGE cohort Readmission or death within 30 days				HOMERuN cohort Readmission or death within 30 days			
	No		Yes		No		Yes	
Total cohort = 62,195	*N* = 57,491 (92.4%)		*N* = 4704 (7.6%)		*N* = 10,320 (91%)		*N* = 1018 (9.0%)	
Age, years								
Mean (SD)	61.3	(16.8)	68.8	(15.5)	58.2	(17.6)	63.9	(17.2)
Sex								
Male	29,637	(51.6%)	2642	(56.2%)	5351	(51.9%)	585	(57.5%)
Race								
White	32,256	(56.1%)	2902	(61.7%)	4724	(46.8%)	521	(51.8%)
Black	10,817	(18.8%)	863	(18.3%)	1730	(17.1%)	152	(15.1%)
Asian	1790	(3.1%)	133	(2.8%)	617	(6.1%)	61	(6.1%)
Other or NA	12,628	(22.0%)	806	(17.1%)	3020	(29.9%)	271	(27.0%)
Ethnicity								
Hispanic	17,323	(30.1%)	1164	(24.7%)	2472	(32.0%)	164	(24.2%)
Smoking status								
Current	210	(0.4%)	23	(0.5%)	550	(5.3%)	80	(7.9%)
Former	10,025	(17.4%)	1109	(23.6%)	1947	(18.9%)	254	(25.0%)
Admission source								
Facility	1419	(2.5%)	156	(3.3%)	382	(3.7%)	64	(6.3%)
Referral	48,922	(85.1%)	3889	(82.7%)	9030	(87.5%)	861	(84.6%)
Other or NA	7150	(12.4%)	659	(14.0%)	908	(8.8%)	93	(9.1%)
Length of stay								
Median (IQR)	5.3	(3.0, 9.3)	4.2	(2.0, 8.1)	5.0	(3.0, 9.2)	6.0	(3.0, 12.0)
Discharge destination								
Home	45,577	(79.3%)	3156	(67.1%)	8454	(81.9%)	682	(67.0%)
SNF/rehab/LTAC	8539	(14.9%)	1120	(23.8%)	1442	(14.0%)	283	(27.8%)
Other or NA	3375	(5.9%)	428	(9.1%)	424	(4.1%)	53	(5.2%)
COVID complications								
VTE	1924	(3.3%)	128	(2.7%)	262	(2.5%)	24	(2.4%)
CHF	7428	(12.9%)	1098	(23.3%)	838	(8.1%)	176	(17.3%)
DIC	88	(0.2%)	4	(0.1%)	8	(0.1%)	2	(0.2%)
AKI	14,205	(24.7%)	1551	(33.0%)	1365	(13.2%)	237	(23.3%)
COVID treatments								
Corticosteroids	45,278	(78.8%)	3777	(80.3%)	2803	(27.2%)	346	(34.0%)
Remdesivir	23,755	(41.3%)	1370	(29.1%)	2419	(23.4%)	184	(18.1%)
Other^a^	17,744	(30.1%)	1147	(24.4%)	220	(2.1%)	43	(4.2%)

^a^Includes convalescent plasma, IL-6 inhibitors, and JAK inhibitors. In the HOMERuN cohort, these data are only available for New York University Hospital
